# Opium in Hemorrhage

**Published:** 1846-12

**Authors:** 


*Opium in Hemorrhage.* By Dr. Griffin.—Of all the wonderful influences exerted by opium, that by which it sustains the powers of life when sinking from hemorrhage, and arrests the flow of blood, is the most extraordinary. When after severe uterine hemorrhage the countenance is sunk, the eye hollow and glassy, the lips blanched, the skin cold, and the whole person corpse-like, when the pulse is almost gone at the wrist, when the beat even of the heart is scarcely perceptible, and stimulants, even brandy or rectified spirits, are either vomited or uninfluential, there remains yet one remedy capable of restoring the patient to life, and that is opium. I believe its power of saving life in these circumstances depends principally on its specific property of producing congestion in the brain. That amount of congestion by which it occasions apoplexy when given in large doses to persons in health, seems only sufficient to sustain the natural and necessary tension of the cerebral vessels in those who are dying of hemorrhage. Persons die in cases of hemorrhage, not so much from mere debility of the heart's action, as from the loss of nervous power in the brain consequent to it, and hence a fainting from which they are never awakened. The opium in such cases not only stimulates the heart's action, but restores a sufficient degree of tension in the vessels of the brain to prevent faintness, and by the judicious repetition of the remedy, life is preserved on the very borders of death. There are no instances in which opium can be given so freely or so fearlessly as in these. When the danger is imminent, five grains may be given at the first dose, and two or three every hour or half hour afterwards, until the pulse becomes distinct, the breathing easier, and tossing and flinging about in the bed is allayed. It is hardly necessary to observe, that in such cases, in conjunction with the use of opium, the administration of warm wine and brandy (however inefficient alone), and the application of heat to the extremities, are highly useful, if not absolutely essential.—*British and Foreign Med. Rev.—Ibid.*

